# Spironolactone in end stage renal disease: A story of under’ACHIEVE’ment

**DOI:** 10.1590/2175-8239-JBN-2025-0270en

**Published:** 2025-11-28

**Authors:** Gerry George Mathew

**Affiliations:** 1 SRM Institute of Science and Technology, SRM Medical College Hospital and Research Centre, Department of Nephrology, Kattankulathur, India.

Dear Editor,

Mineralocorticoid receptor (MR) activation affects distal renal tubules, sweat glands, salivary glands, cardio­myocytes, podocytes, fibroblasts, and endothelial cells. MR antagonists are vital in chronic kidney disease (CKD) due to their anti-inflammatory and antifibrotic effects^1^. Aldosterone has a pivotal role in promoting left ventricular hypertrophy (LVH), increasing left ventricular mass index (LVMI) and advancing coronary artery disease (CAD) and myocardial fibrosis^1^. Spironolactone is a non-selective MR antagonist affecting androgen and progesterone receptors^1^. MR antagonists reduce proteinuria, stabilize estimated glomerular filtration rate, and regress left ventricular hyper­trophy in CKD stages 1–4^1,2^. These benefits led investigators to study cardiovascular benefits in hemodialysis patients from the ACHIEVE^3^ and ALCHEMIST^4^ trials.

The ACHIEVE^3^ trial was a multi-center randomized controlled trial of spironolactone versus placebo conducted at 143 dialysis centers across 12 countries. After a 7- to 14.5-week run-in phase, 2538 patients were assigned to 25 mg spironolactone (n = 1260) and placebo (n = 1278)^3^. The primary outcome was time to first composite of cardiovascular death or heart failure hospitalization. During the 1.8 years median follow-up, the primary outcome occurred in 258 patients of the spironolactone group (10 events/100 patient-years) and in 276 patients of the placebo group (11 events/100 patient-years), with 0.92 (95% CI 0.78–1.09) hazard ratio^3^. The trial was stopped for futility. Spironolactone showed 50% higher severe hyperkalemia rates despite excluding 370 participants for hyperkalemia during run-in^3^. In the treatment group, 294 patients discontinued, while 241 in the placebo group stopped, mainly due to patient preference and hospitalizations^3^.

Several design issues undermine the trial’s validity. The sample size calculation was adjusted from 2,648 participants to 650 events, suggesting insufficient initial power. The study included a run-in period of 7 to 14.5 weeks requiring adherence to spironolactone, which introduced selection bias as 28% of participants were not randomized. The 1–2-year time constraints are inadequate given ESRD complications and the early termination due to futility, which limited detection of cardiovascular benefits. The effects of mineralocorticoid receptor antagonists on cardiac remodeling typically require prolonged treatment^5^, making short-term MRA use unlikely to show improvement. Furthermore, addressing the hyper-aldosterone condition after starting dialysis, when significant left ventricular mass and extensive cardiac fibrosis are already present, is unlikely to show improvement with short-term use of MRA^5^.

The composite primary outcome combining cardiovascular death with heart failure hospitalizations may dilute treatment effects across international sites. Confounding factors include diverse dialysis populations with different prescriptions, medications, and potassium management strategies. The optional use of potassium binders and dialysate adjustments introduced variability. Treatment adherence presents limitations, as self-reported adherence during the run-in phase was 80% and the option to reduce dosing to three times a week or temporarily stop the study medication further complicates the interpretation of the actual treatment effects. Despite the rigorous design of the ALCHEMIST^4^ trial evaluating spironolactone in hemodialysis patients, methodological constraints—including sample size, selection bias, follow-up duration, endpoint choice, and safety monitoring intensity—influence the clinical applicability of these findings.

Based on current evidence, spironolactone shows no role in reducing cardiovascular mortality and heart failure hospitalization in hemodialysis patients.

## Data Availability

The data for substantiating the findings of this study are available with the corresponding author and can be made available on request.
