# Supplementary material for: Spironolactone in end stage renal disease: A story of under’ACHIEVE’ment
Source: J Bras Nefrol. 2025 Nov 28;48(1):e20250270. doi: 10.1590/2175-8239-JBN-2025-0270en (PMC12674906; doi:10.1590/2175-8239-JBN-2025-0270en)
Supplement: Translation

# Translation: Espironolactona na doença renal em estágio terminal: uma história de “desempenho abaixo do esperado”

**DOI:** 10.1590/2175-8239-JBN-2025-0270pt

**Authors:** Gerry George Mathew

**Affiliations:** 1 SRM Institute of Science and Technology, SRM Medical College Hospital and Research Centre, Department of Nephrology, Kattankulathur, Índia.

Caro Editor,

A ativação do receptor mineralocor­ticoide (RM) afeta os túbulos renais distais, as glândulas sudoríparas, as glândulas salivares, os cardiomiócitos, os podócitos, os fibroblastos e as células endoteliais. Os antagonistas do RM têm grande importância na doença renal crônica (DRC) devido aos seus efeitos anti-inflamatórios e antifibróticos^1^. A aldosterona desempenha um papel fundamental na promoção da hipertrofia ventricular esquerda (HVE), no aumento do índice de massa ventricular esquerda (IMVE) e no avanço da doença arterial coronariana (DAC) e da fibrose miocárdica^1^. A espironolactona é um antagonista não seletivo do RM que afeta os receptores de androgênio e pro­gesterona^1^. Os antagonistas do RM reduzem a proteinúria, estabilizam a taxa de filtração glomerular estimada e promovem regressão da hipertrofia ventricular esquerda nos estágios 1 a 4 da DRC^1,2^. Esses efeitos positivos levaram os pes­quisadores a estudar os benefícios cardiovasculares em pacientes em hemodiálise a partir dos estudos ACHIEVE^3^ e ALCHEMIST^4^.

O estudo ACHIEVE^3^ foi um ensaio clínico multicêntrico, randomizado e controlado, que comparou espironolactona e placebo, realizado em 143 centros de diálise distribuídos por 12 países. Após uma fase de *run-in* de 7 a 14,5 semanas, 2.538 pacientes foram designados para receber 25 mg de espironolactona (n = 1.260) ou placebo (n = 1.278)^3^. O desfecho primário foi o tempo até o primeiro evento composto de morte cardiovascular ou hospitalização por insuficiência cardíaca. Durante o acompanhamento mediano de 1,8 anos, o desfecho primário ocorreu em 258 pacientes do grupo espironolactona (10 eventos/100 pacientes-ano) e em 276 pacientes do grupo placebo (11 eventos/100 pacientes-ano), com razão de risco de 0,92 (IC 95% 0,78–1,09)^3^. O ensaio foi interrompido por futilidade. A espironolactona apresentou taxas 50% mais elevadas de hipercalemia grave, apesar da exclusão de 370 participantes por hipercalemia durante o período de *run-in* ^3^. No grupo de tratamento, 294 pacientes descontinuaram o uso do medicamento, enquanto 241 pacientes do grupo placebo interromperam o tratamento devido, principalmente, à preferência dos pacientes e a hospitalizações^3^.

Diversas questões relacionadas ao desenho do estudo comprometem a validade do ensaio. O cálculo do tamanho da amostra foi ajustado de 2.648 participantes para 650 eventos, sugerindo poder inicial insuficiente. O estudo incluiu um período de *run-in* de 7 a 14,5 semanas, exigindo adesão à espironolactona, o que introduziu viés de seleção, uma vez que 28% dos participantes não foram randomizados. As restrições de tempo de 1 a 2 anos mostraram-se inadequadas, considerando as complicações da DRET e a interrupção precoce em razão da futilidade, o que limitou a detecção de benefícios cardiovasculares. Os efeitos dos antagonistas do receptor mineralo­corticoide (ARM) na remodelação car­díaca normalmente requerem tratamento prolongado^5^, tornando improvável que o uso de ARM em curto prazo apresente melhora. Além disso, abordar a condição de hiperaldosteronismo após o início da diálise, quando já existem massa ventricular esquerda significativa e fibrose cardíaca extensa, provavelmente não apresentará melhora com o uso de ARM em curto prazo^5^.

O desfecho primário composto, que combinou morte cardiovascular e hospitalizações por insuficiência cardíaca, pode ter diluído os efeitos do tratamento entre os centros internacionais. Os fatores de confusão incluíram populações de diálise diversas, com diferentes prescrições, medicamentos e estratégias de manejo de potássio. O uso opcional de quelantes de potássio e os ajustes no dialisato introduziram variabilidade. A adesão ao tratamento apresentou limitações, uma vez que a adesão autorrelatada durante a fase de *run-in* foi de 80% e a opção de reduzir a dosagem para três vezes por semana ou interromper temporariamente a medicação do estudo dificultou ainda mais a interpretação dos efeitos reais do tratamento. Apesar do desenho rigoroso do estudo ALCHEMIST^4^, que avaliou o uso da espironolactona em pacientes em hemodiálise, as restrições metodológicas — incluindo tamanho da amostra, viés de seleção, duração do acompanhamento, escolha dos desfechos e intensidade do monitoramento de segurança — influenciam a aplicabilidade clínica desses achados.

Com base nas evidências atuais, a espironolactona não demonstra ter qualquer papel na redução da mortalidade cardiovascular e das hospitalizações por insuficiência cardíaca em pacientes em hemodiálise.

## Data Availability

Os dados que fundamentam os achados deste estudo estão disponíveis com o autor correspondente e podem ser disponibilizados mediante solicitação.
